# Intravenous Injection of Collargolum in Puerperal Fevcr

**Published:** 1906-11

**Authors:** Paul Hocheisen

**Affiliations:** Staff Physician, detailed as Assistant at the University Gynecological Clinic of the Royal Charite Hospital at Berlin


### ABSTRACTS.

### Intravenous Injection of Collargolum in Puerperal Fevcr.

By DR. PAUL HOCHEISEN,

Staff Physician, detailed as Assistant at the University Gynecological Clinic of the Royal Charite Hospital at Berlin.

[*Medicinische Klinik, Nos. 31—4, 1906.*]

HOCHEISEN'S exhaustive study of collargolum in puerperal fever (*Medicinische Klinik,* Nos. 31|4, 1906) opens with a review of its literature in this field. This is overwhelmingly favorable, though the effect from collargolum was in many cases subjective only. Subjective improvement, however, is an important criterion of the value of a remedy for general septic disease; these infections run an extremely varied course and the prognosis is not infrequently an opinion based on subjective symptoms. Especially is this true in the class of sepsis known as puerperal fever, which is no clinical entity and includes processes that differ widely, both etiologically and symptomatically.

The author details the histories of 52 cases which he had in the Charité during two years, dividing them into the following groups: I. Sapremic and septic endometritis. II. Phlegmasia alba dolens. III. Pyemia. IV. Septicemia. V. Metritis dissecans. VI. Adnexal tumors and parametritis. VII. Peritonitis.

While in most cases of sapremic and septic endometritis the effect on the temperature was not very marked, the pulse usually became slower and stronger and the general condition always improved. In phlegmasia alba dolens there also was general betterment, and the chills ceased so suddenly when collargolum treatment was instituted that the effect must be ascribed to the remedy.

In the 13 cases of pyemia three died; but two of the fatal cases were moribund on admission and the third was hopeless from the first. Six of the cured cases were of the severest type and their recovery was undoubtedly due to collargol. All other resources were of course also used, the use of stomachics playing an important role; but collargol proved to be the very best stomachic,—a fact which demanded recognition again and again. Perhaps the most important immediate effect was the subjective improvement. The effect is best seen in the worst cases, with pneumonic processes and endocarditic ulcerations. Here its action is sometimes fairly marvelous and cannot be justly depicted in the histories. These cases were formerly almost always fatal.

All the septicemia cases died; nor did collargol seem to have any effect in the three in which it was tried. In metritis dissecans, collargol cannot of course influence the process in the uterine muscle; the utmost it can do is to combat the general infection.

As to adnexal tumors and parametritis, Hocheisen cannot confirm the resorptive action of the drug on infiltrations and suppurative foci that some observers recorded. When fever is high, collargol is beneficial by its antifebrile effect and favorable influence on the general condition. It is most valuable, however, before localisation of the process occurs and when general infection is threatening or already present. It may also be that it hastens localisation. In three peritonitis cases in which collargol was used it did not show any effect.

The puerperal mortality in the Charité during the two collargol years was much below the mortality in any other year of the past decade, especially if only the pyemia, septicemia and peritonitis cases are considered. In these the mortality in the collargol years is 27% less than the ten-year average; it is 19% less than in the best other year and 45% less than in the worst. Nor were the collargol cases lighter than usual; rather can it be said that good results were attained despite unusually severe types. The pyemia cases gave a mortality of 23% as against the ten-year average of 58%. The timely intravenous injection of collargol would save many otherwise fatal cases.

Hocheisen concludes: Collargol, while not a specific, is an invaluable aid in suitable cases. It diminishes the temperature, stops the chills, effects subjective and objective improvement, induces a welcome diaphoresis, stimulates appetite and produces sleep. Very often it diminishes the pulse-rate and regulates blood-pressure. Its special domain is pyemia, where it minimises the mortality. Endocarditis and septic pneumonia seem especially susceptible to its influences; the finding of collargol on the ulcerated cardiac valve in one of the autopsies perhaps points out the way in which it acts on septic food and thrombi reached by the blood current. Possibly it inhibits the decomposition of thrombi; probably it favors the localisation of processes that have extended beyond the uterus; certainly it combats the progress of the general infection.

It is, therefore, indicated when there are signs of a beginning general infection and as long as thorough localisation of the disease process has not occurred. Three to five cc. (45 to 75 m.) of a 2% collargol solution or smaller amounts of stronger (up to 5%) solutions should be injected intravenously on two or three consecutive days. This usually suffices, but the injections can be repeated as desired. The technique is simple; if the insertion of the needle into the vein through the stretched skin does not succeed, the vessel is laid bare.

The injection is painless; if pain is felt or the skin at the site turns blue, it has been subcutaneous, is inefficacious and occasions infiltration. Quite recently Privy Councillor Dr. Bumm had two cases of grave pyemia in his division treated with collargol, and their favorable course confirms Hocheisen's observations.

